# Vitamin D deficiency and clinical correlations in systemic sclerosis patients: A retrospective analysis for possible future developments

**DOI:** 10.1371/journal.pone.0179062

**Published:** 2017-06-09

**Authors:** Amelia Chiara Trombetta, Vanessa Smith, Emanuele Gotelli, Massimo Ghio, Sabrina Paolino, Carmen Pizzorni, Amber Vanhaecke, Barbara Ruaro, Alberto Sulli, Maurizio Cutolo

**Affiliations:** 1 Research Laboratory And Academic Division Of Clinical Rheumatology, Department Of Internal Medicine, Irccs San Martino Aou, University Of Genoa, Genoa, Italy; 2 Department Of Rheumatology, Ghent University Hospital, Ghent University, Ghent, Belgium; University of Alabama at Birmingham, UNITED STATES

## Abstract

**Objective:**

Assessment of serum 25-hydroxyvitamin D (25(OH)D) correlations with clinical parameters and evaluation of the efficacy of standard oral supplementation in systemic sclerosis (SSc) patients.

**Methods:**

154 SSc patients were recruited, in all seasons. Serum 25(OH)D concentrations were evaluated using LIAISON 25-OH (Diasorin, Italy). Medsger disease severity scale (DSS), nailfold videocapillaroscopy (NVC) and all instrumental exam contemplated by international guidelines were performed. Drug assumption, including oral colecalciferol, was evaluated. Non-parametric tests were used for statistical analysis.

**Results:**

Average 25(OH)D serum concentration was 18.7 ±9 ng/ml (<20 classified as deficiency). A significant correlation was found with presence/absence of lung bi-basal fibrotic changes (16.1 ±8 ng/ml and 20 ±10 ng/ml, respectively; p = 0.04). Peripheral vascular (p = 0.03), kidney (p = 0.02), gastrointestinal (p = 0.05) Medsger’s DSS parameters were found to correlate with 25(OH)D serum concentrations. No significant correlations were observed with digital ulcers incidence, strictly correlated to patterns of microangiopathy, defined at NVC analysis (p<0.0001). Interestingly, no effects of treatment with oral colecalciferol (Dibase 1,000 IU daily for at least 6 months) were found on 25(OH)D serum concentrations in treated (18.8 ±10 ng/ml) or untreated (18.7 ±9 ng/ml) SSc patients (p = 0.81). A significant difference was observed among seasonal 25(OH)D serum concentrations (winter: 14.6 ±7.8 ng/ml, spring: 17.2 ±7.9 ng/ml, summer: 21.43 ±10 ng/ml, autumn: 20.2 ±10 ng/ml; p = 0.032) in all patients.

**Conclusion:**

Serum 25(OH)D deficiency was found to correlate with lung involvement, peripheral vascular, kidney and gastrointestinal Medsger’s DSS parameters and with seasonality In SSc patients. Supplementation with oral colecalciferol was found not effective in increasing 25(OH)D serum concentrations. Therefore, for successful replacement, supra-physiological vitamin D3 doses or programmed UVB light exposure should be tested.

## Introduction

Systemic sclerosis (SSc) is a connective tissue disease of multifactorial etiology, mainly characterized by microvascular damage followed by progressive fibrosis of skin and internal organs [1]. In SSc patients, low 25-hydroxyvitamin D (25(OH)D) serum concentrations have been shown and 25(OH)D deficiency was demonstrated to interfere with immune response [2, 3].

Vitamin D seems to play a key role both on innate and adaptive immune system and the presence of its receptors on different cells types, seems to support this hypothesis. Especially in systemic lupus erythematosus (SLE) and rheumatoid arthritis, a deficit in vitamin D serum concentrations is evident and seems to correlate with disease course and outcome [4, 5].

Arnson et al. demonstrated, in a large multinational SSc patients’ population, that lower serum vitamin D concentrations were inversely correlated with the extent of cutaneous fibrosis. However, the effects of vitamin D supplements and seasonality of clinical assessments were not evaluated [6].

The purpose of the study was to correlate 25(OH)D serum concentrations, in a large cohort of SSc patients from two European referral centers, with clinical complications and seasonality. A further aim was to evaluate the efficacy of supplementation with oral colecalciferol in increasing 25(OH)D serum concentrations.

## Methods

**Fig 1:**
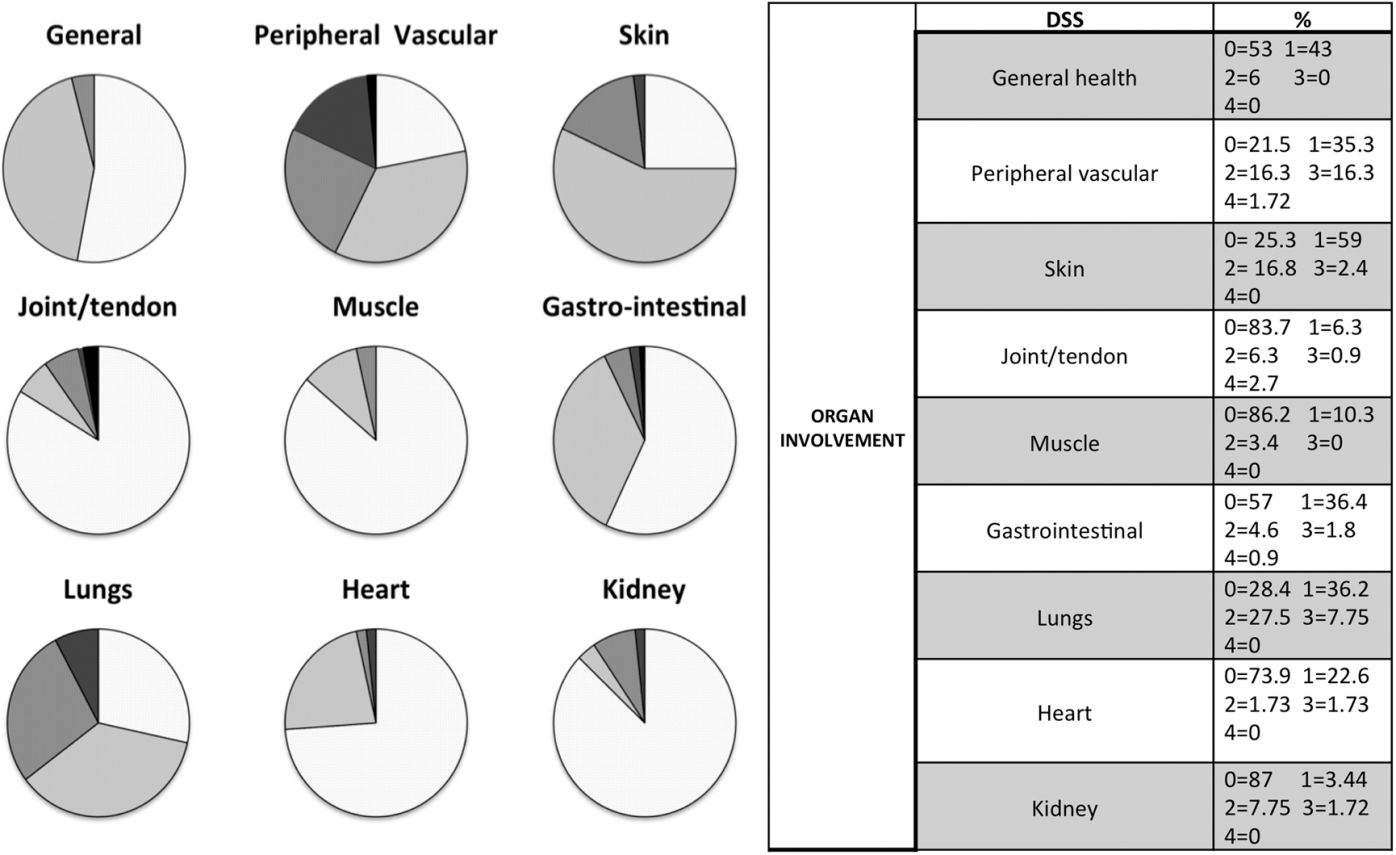
Medsger’s disease severity scale (DSS) parameters in the study population. In the left part of the figure, a graphic representation describes organ involvement through the distribution of the five values (0 to 4) for each parameter of Medsger’s DSS (white = none, light grey = mild, intermediate grey = moderate, dark grey = severe, black = end-stage). In the right part of the figure the same distribution is described in percentages (0 = none, 1 = mild, 2 = moderate, 3 = severe, 4 = end-stage).

A population of 154 consecutive SSc patients (diagnosis based on LeRoy 2001 and later confirmed by 2013 American College of Rheumatology (ACR)/European League Against Rheumatism (EULAR) criteria) was enrolled in the study [7, 8]). 91 patients were followed up at the Rheumatology Division of Genoa University (Italy) and 63 at the Department of Rheumatology of Ghent University Hospital (Belgium). As part of the regular follow up approved by both Institutions for SSc, all patients undergone clinical evaluation and assessment of disease severity through the disease severity scale (DSS), developed by Medsger at al [9] (Fig 1). Serum concentration of 25(OH)D was evaluated by the direct competitive chemi-luminescence DiaSorin LIAISON immunoassay (normal: 30–100 ng/ml). A concentration between 20 and 30 ng/ml was classified as 25(OH)D insufficiency, while concentrations <20 ng/ml referred as deficiency [10]. In all patients calcium concentrations were determined through COBAS, Roche Diagnostics Ltd, (normal: 8.5–10.5 mg/dl). In 44 patients also PTH serum concentrations was determined through DiaSorin LIAISON immunoassay (normal: 6.5–36 ng/l).

Patients assuming oral cholecalciferol (Dibase Abiogen Pharma, Italy), at the recommended dose of 1,000 IU per day, for hypovitaminosis D prevention, were included in the study.

On the same date, nailfold videocapillaroscopy (NVC) was performed in all patients by the same operator using an optical probe, equipped with a 200× contact lens, connected to an image analysis software (Videocap, DS Medica, Milan, Italy). Nailfold capillaries abnormalities were scored by a validated qualitative and semi-quantitative rating scale, in accordance with previous studies [1, 11–13].

Pulmonary function test with diffusing capacity of the lungs for carbon monoxide (DLCO), chest x-ray, lung CT scan, electrocardiography (ECG), Doppler echocardiography with systolic pulmonary arterial pressure (sPAP) measurement, renal artery resistive index (RI) by echo color Doppler, were performed. Dual X-ray absorptiometry (DXA) was performed annually, according to National Osteoporosis Foundation guidelines, to calculate trabecular bone score (TBS) and bone mineral density (BMD) at the lumbar spine, hip, and proximal femur [14]. Drug assumption related effects were also evaluated (S1 File). All patients were informed that their data would be used for possible studies/analyses and gave their consent when starting to be followed up at the Clinic. Data were analyzed using IBM SPSS Statistics, Version 21.0. (IBM Corp: Armonk, NY). Non-parametric tests were used for statistical analysis. A p value <0.05 was considered significant.

## Results

### Patients

Of the 154 patients enrolled, 131 were women and 21 men. Age was 59 ±15 years. Average Raynaud’s phenomenon (RP) duration was 13.4 ±12.7 years, average disease duration was 6.5 ±6.3 years (Table 1). Limited cutaneous (lcSSc) form frequency was 60%, diffuse cutaneous form (dcSSc) frequency was 25%, and limited SSc (LSSc) form frequency was 15% (Table 1).

**Table 1:** The table describes patients’ clinical characteristics, starting from personal data (age and gender), disease specific characteristics (disease subtype in percentages, autoantibody positivity in percentages, Raynaud’s phenomenon and disease durations, general digital ulcer incidence and according to pattern of microangiopathy).

Patients clinical characteristics		TOT 154	
**Age (years)**		59 ±15	
**Gender (%)**		W = 131 (85)	
		M = 23 (15)	
**Disease subtype (%)**	**dcSSc**	25	
	**lcSSc**	60	
	**LSSc**	15	
**Autoantibody positivity (%)**	**Anti-Scl 70 Ab**	28.2	
	**ACA Ab**	56.4	
**Average RP duration (years)**		13.4 ±12.7	
**Average disease duration (years)**		6.5 ±6.3	
**DUs incidence (%)**		32	
**By NVC pattern of micro-angiopathy**		“Early”: 8	(p<0.0001)
		“Active”: 33	
		“Late”: 34	
**Average 25(OH)D (ng/ml)**		18.7 ±9	
**Average 25(OH)D by region (ng/ml)**		Italy: 18 ±10	(p = 0.99)
		Belgium: 19 ±9	
**Average 25(OH)D by season (ng/ml)**		Winter: 14.6 ±7.8	(p = 0.032)
		Spring: 17.2 ±7.9	
		Summer: 21.43 ±10	
		Autumn: 20.2 ±10	
**Calcium (mg/dl)**		9.3 ±0.4	
**PTH (44 patients) (ng/l)**		28.6 ±14	
	**Vit D supplementation**	22	
**Treatments**	**Glucocorticoids**	18.1	
**(%)**	**Calcium Channel blockers**	22	
	**Cyclic intravenous iloprost**	61	
	**PAH inhibitors**	7.8	

25(OH)D serum levels are reported as mean ± standard deviation and p value for all patients, according to patients region of origin and according to season in which the patient has been evaluated). PTH serum levels are reported as mean ± standard deviation. Treatments regimens considered relevant were reported as percentages of patients’ population. W = women; M = men; DcSSc = diffuse cutaneous systemic sclerosis; lcSSc = limited cutaneous systemic sclerosis; LSSc = limited systemic sclerosis; Anti-Scl 70 Ab = anti–topoisomerase I antibody; ACA = anti-centromere antibody; RP = Raynaud’s phenomenon; DUs = digital ulcers; NVC = Nailfold Videocapillaorscopy; PAH = pulmonary artery hypertension.

There was no statistically significant correlation between 25(OH)D serum concentrations and sex (women 18.5 ±10 ng/ml, men 19.61 ±9 ng/ml; p = 0.63), age (p = 0.81), RP duration (p = 0.69), disease duration (p = 0.43), dcSSc/lcSSc/LSSc involvement (dcSSc = 17 ±10 ng/ml, lcSSc 17 ±8 ng/m, LSSc 20 ±10 ng/ml; p = 0.49).

Thirty-two percent of patients had digital ulcers (DUs) at the time of the investigation. No correlation was reported between DUs incidence and 25(OH)D serum concentrations (no DUs = 20.5 ±11 ng/ml, DUs = 15.5 ±10 ng/ml; p = 0.13), while DUs incidence correlated significantly with patterns of microangiopathy (8% in “Early”, 33% in “Active”, 34% in “Late” patients; p<0.0001). Morever, sub-classifying together “peripheral vascular” disease severity scale parameter 0 to 1 (absence of digital trophic lesions) and 2 to 4 (presence of digital trophic lesions), no significant difference was observed in 25(OH)D serum concentrations: 0–1 group = 18.3 ±9 ng/ml; 2–4 group = 17.1 ±9 ng/ml (p = 0.54). A highly significant correlation with patterns of microangiopathy persisted in the last classification (digital trophic lesions 25% in “Early”, 43% in “Active”, 63% in “Late” patients; p<0.0001).

Relevant treatment regimens are reported in Table 1.

### Laboratory and instrumental variables

Average 25(OH)D serum concentration in SSc patients was found 18.7 ±9 ng/ml (Table 1). Of the 154 patients analyzed, 124 (80.5%) had 25(OH)D serum concentrations ≤30 ng/ml, i.e. classifiable as insufficiency, 87 (56.5%) ≤20 ng/ml, defined as deficiency [11].

Anti-centromere antibodies **(ACA-Ab)** were detectable in 56.4%, Anti-Scl70 Ab in 28.2% of SSc patients (Table 1). The average instrumental variables values are reported in Table 2.

No regional/latitude difference was observed between 25(OH)D serum concentrations in the two groups: patients from Italy had an average of 18 ±10 ng/ml, patients from Belgium had an average of 19 ±9 ng/ml (p = 0.99) (Table 1).

**Table 2:** The table describes organ involvement in patients population as assessed through laboratory (creatinine levels) and instrumental parameters: Average pulmonary function test measurements (DLCO%, FVC% and FVC%/DLCO% ratio), average echocardiographic estimation of SPAP values, patients percentage showing interstitial lung disease or bi-basal fibrotic changes at lung CT scan, patients percentage showing enlarged hearth at chest X-rays, patients percentage showing ECG alterations (conduction disorders and arrhythmias), patients percentage for each nailfold videocapillaroscopic pattern.

Laboratory and instrumental parameters			TOT 154
**Blood tests**	Creatinine levels (mg/dl)		1.01 ±0.4
**Pulmonary**	DLCO % (mean ±SD)		75 ±18
**function**	FVC% (mean ±SD)		104 ±21
**test**	FVC%/DLCO% (mean ±SD)		1.4 ±0.4
**Echocardiography**	sPAP (mean ±SD)		31.6 ±7
**CT Scan**	Interstitial lung disease (%)		46
	Bi-basal fibrotic changes (%)		44
**Chest X-rays**	Enlarged heart (%)		12
**Electrocardiography**	Conduction disorder (%)		19
	Arrythmias (%)		5
**NVC**	Patterns (%)	Early	15.6
		Active	34.3
		Late	50

DLCO = diffusing capacity of the lungs for carbon monoxide; SD = standard deviation; FVC = forced vital capacity, sPAP = systolic pulmonary arterial pressure, NVC = Nailfold Videocapillaroscopy.

**Fig 2:**
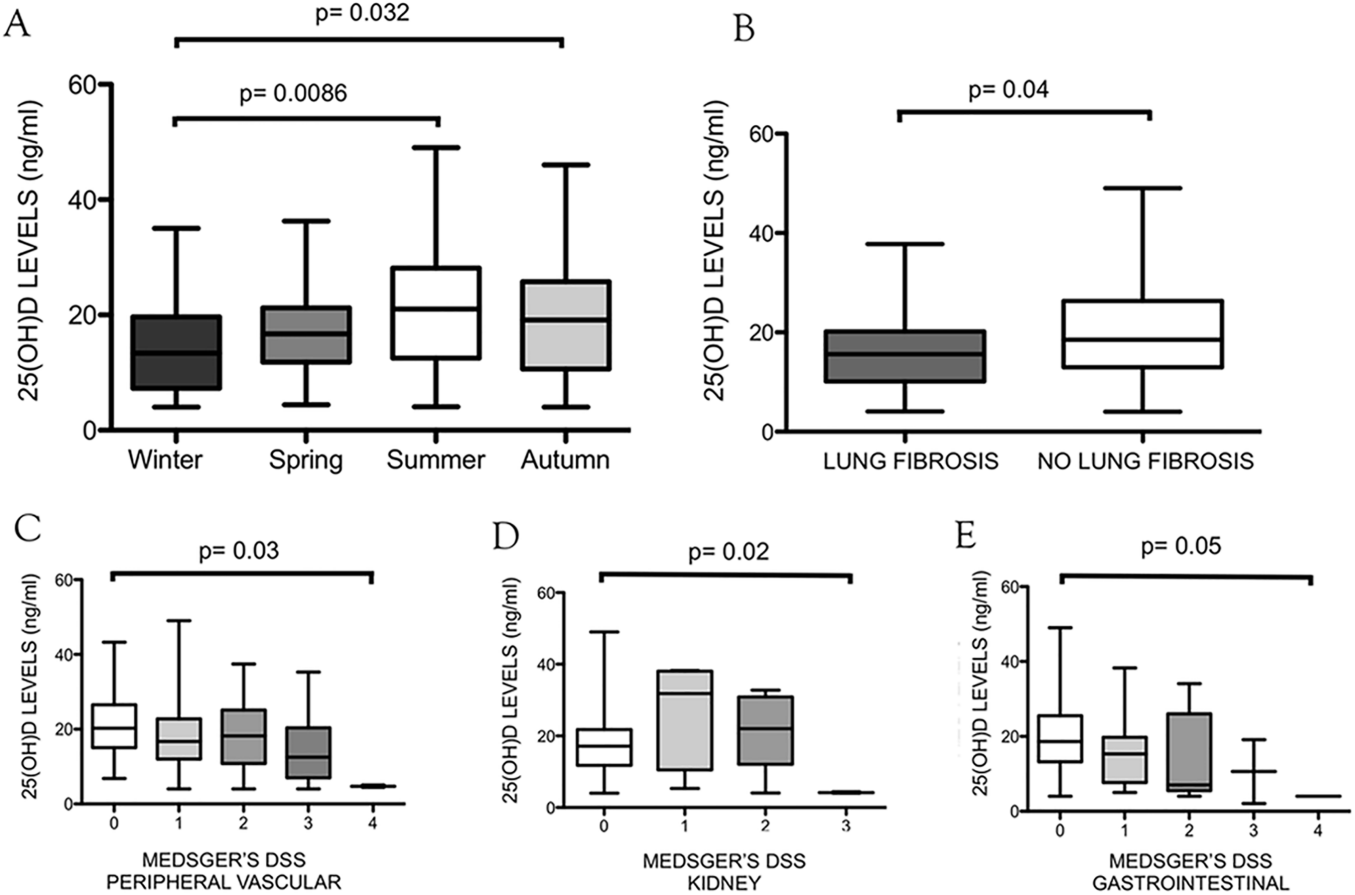
25(OH)D serum levels and significant correlations. A) Average 25(OH)D serum concentrations in all seasons: a statistically significant difference was observed among seasonal 25(OH)D serum concentrations (p = 0.032). The Dunn’s multiple comparison post-test shows a more significant difference between patients observed in winter compared to summer months (p = 0.0086). B) Average 25(OH)D serum concentrations in patients showing presence/absence of bi-basal fibrotic changes at lung CT scan: a statistically significant difference was found between 25(OH)D serum concentrations in patients showing presence vs absence of bi-basal fibrotic changes at lung CT scan (p = 0.04). C) Medsger’s disease severity scale “peripheral vascular” parameter correlation with 25(OH)D serum concentrations (p = 0.03).D) Medsger’s disease severity scale “kidney” parameter correlation with 25(OH)D serum concentrations (p = 0.02). E) Medsger’s disease severity scale “gastro-intestinal” parameter correlation with 25(OH)D serum concentrations (p = 0.05).

A significant difference was observed among seasonal vitamin D concentrations in the population as a whole (p = 0.032). The Dunn’s multiple comparison post-test showed a more significant difference between patients observed in winter compared to summer months (p = 0.0086) (Table 1 and Fig 2A).

Average calcium concentrations were determined in all patients: 9.3 ±0.4 mg/dl. In 44 patients also PTH serum concentrations were determined: 28.6 ±14 ng/l (Table 1). A tendency to direct correlation was observed between 25(OH)D and calcium serum concentrations (p = 0.050, Spearman r = 0.18) while a non significant tendency to inverse correlations was observed between 25(OH)D and PTH serum concentrations (p = 0.07, Spearman r = -0.27).

Interestingly, a statistically significant correlation was found between 25(OH)D serum concentrations and presence/absence of bi-basal fibrotic changes at lung CT scan (average: 16.1 ±8 ng/ml and 20 ±10 ng/ml, respectively, p = 0.04) (Fig 2B).

No correlation was found between 25(OH)D serum concentrations and autoantibody specificity (anti-centromere Ab = 18.5 ±10 ng/ml, Scl-70 Ab 18.9 ±9 ng/ml; p = 0.84), videocapillaroscopic pattern (“Early” = 23.3 ±11 ng/ml, “Active” = 18.7 ±9 ng/ml, “Late” = 17.7 ±9 ng/ml; p = 0.2), haemoglobine (Hb) (p = 0.9), erythrocyte sedimentation rate (ESR) (p = 0.37), C-reactive protein (CRP) (p = 0.67), creatinine (p = 0.11), interstitial lung disease at CT scan (presence = 18 ±9 ng/ml, absence = 18 ±11 ng/ml, p = 0.82), total lung capacity (p = 0.23), forced vital capacity (p = 0.7), forced expiratory volume in the 1st second (p = 0.5), DLCO% (p = 0.3), sPAP (p = 0.79) enlarged hearth size at chest x-ray (presence = 18.3 ±10 ng/ml, absence = 18.4 ±10 ng/ml, p = 0.84), conduction disorders at ECG (presence = 20 ±8 ng/ml, absence = 19 ±10 ng/ml, p = 0.3).

No correlations were observed between 25(OH)D serum concentrations and trabecular bone score (TBS) (p = 0.85), BMD, T-scores and Z-scores. Moreover, no correlation was observed among DXA scan data and glucocorticoid therapy (Table 3).

**Table 3:** In the left part of the table DXA scan parameters average values in all patients population are shown. In the right part of the table there are p values for correlation of DXA scan data with 25(OH)D serum levels and with glucocorticoid therapy.

DXA Scan Data		Mean ± SD	Correlation with	Correlation with
			serum 25(OH)D	glucocorticoid therapy
			(p values)	(p values)
L1-L4	BMD	0.9 ±0.17	0.36	0.14
	T SCORE	-1.5 ±1.4	0.83	0.13
	Z SCORE	0.2 ±1.2	0.87	0.36
Neck	BMD	0.7 ±0.1	0.38	0.98
	T SCORE	-2 ±0.9	0.43	0.47
	Z SCORE	-0.5 ±0.8	0.19	0.6
Ward	BMD	0.5 ±0.1	0.38	0.46
	T SCORE	-2.7 ±0.8	0.83	0.44
	Z SCORE	-0.7 ±0.7	0.41	0.7
Trochanteric	BMD	0.6 ±0.1	0.39	0.33
	T SCORE	-1.1 ±0.9	0.51	0.16
	Z SCORE	-0.3 ±0.8	0.50	0.18
Whole	BMD	0.8 ±0.1	0.48	0.24
	T SCORE	-1.6 ±0.9	0.56	0.14
	Z SCORE	-0.3 ±0.8	0.41	0.21

No correlations were observed between 25(OH)D serum concentration and bone mineral density (BMD) at the lumbar spine (L1-L4), hip, and proximal femur. No correlations were observed among dual X-ray absorptiometry (DXA) scan data and glucocorticoid therapy assumption.

Interestingly, an ongoing pilot study shows no correlation between 25(OH)D serum concentration and peripheral blood flow in SSc patients as analyzed by laser speckled contrast analysis (LASCA) or skin thickness, evaluated by skin echography on a small proportion of the same SSc patients (23/154) (data not shown).

### 25-hydroxyvitamin D serum concentrations and Medsger’s disease severity scale parameters

Medsger’s disease severity scale values are reported in Fig 1. DSS parameters that significantly correlated with 25(OH)D serum concentration were: “peripheral vascular” (0 = 21 ±8 ng/ml, 1 = 17.7 ±9 ng/ml, 2 = 18.7 ±9 ng/ml, 3 = 14.99 ±9 ng/ml, 4 = 4.7 ±0.4 ng/ml; p = 0.03), “kidneys” (0 = 17.5 ± 8 ng/ml, 1 = 26.8 ± 15 ng/ml, 2 = 21 ± 10 ng/ml, 3 = 4.2 ± 0.2 ng/ml; p = 0.02), “gastrointestinal” (0 = 19.53 ± 9 ng/ml, 1 = 16.01 ± 9 ng/ml, 2 = 14.00± 12 ng/ml; p = 0.05) (Figs 1, 2C, 2D and 2E).

DSS parameters that did not correlate with 25(OH)D serum concentrations were: “general health” (0 = 8.2 ±9 ng/ml, 1 = 18.41 ±8 ng/ml, 2 = 16.35±14 ng/ml; 0.8), “skin” (0 = 19.8 ±8 ng/ml, 1 = 19.2 ±10 ng/ml, 2 = 17.1 ±9 ng/ml, 3 = 15.3 ±9 ng/ml; p = 0.36), “hearth” (0 = 18.11 ±10 ng/ml, 1 = 19.27 ± 8 ng/ml, 2 = 22.85 ±14 ng/ml, 3 = 19.35 ±9 ng/ml; p = 0.77), “lung” (0 = 17.05 ±8 ng/ml, 1 = 19.9 ±10 ng/ml, 2 = 16.00 ±9 ng/ml, 3 = 18.60 ±8 ng/ml; p = 0.34), “muscle” (0 = 18.1 ±9 ng/ml, 1 = 16.5 ±8 ng/ml, 2 = 18.3 ±9 ng/ml; p = 0.93), “joint/tendon” (0 = 18.18 ±9 ng/ml, 1 = 14.90 ±6 ng/ml, 2 = 17.75 ±9 ng/ml, 3 = 35 ±10 ng/ml, 4 = 18 ±15 ng/ml; p = 0.57).

### 25-hydroxyvitamin D serum concentration and treatments

**Fig 3:**
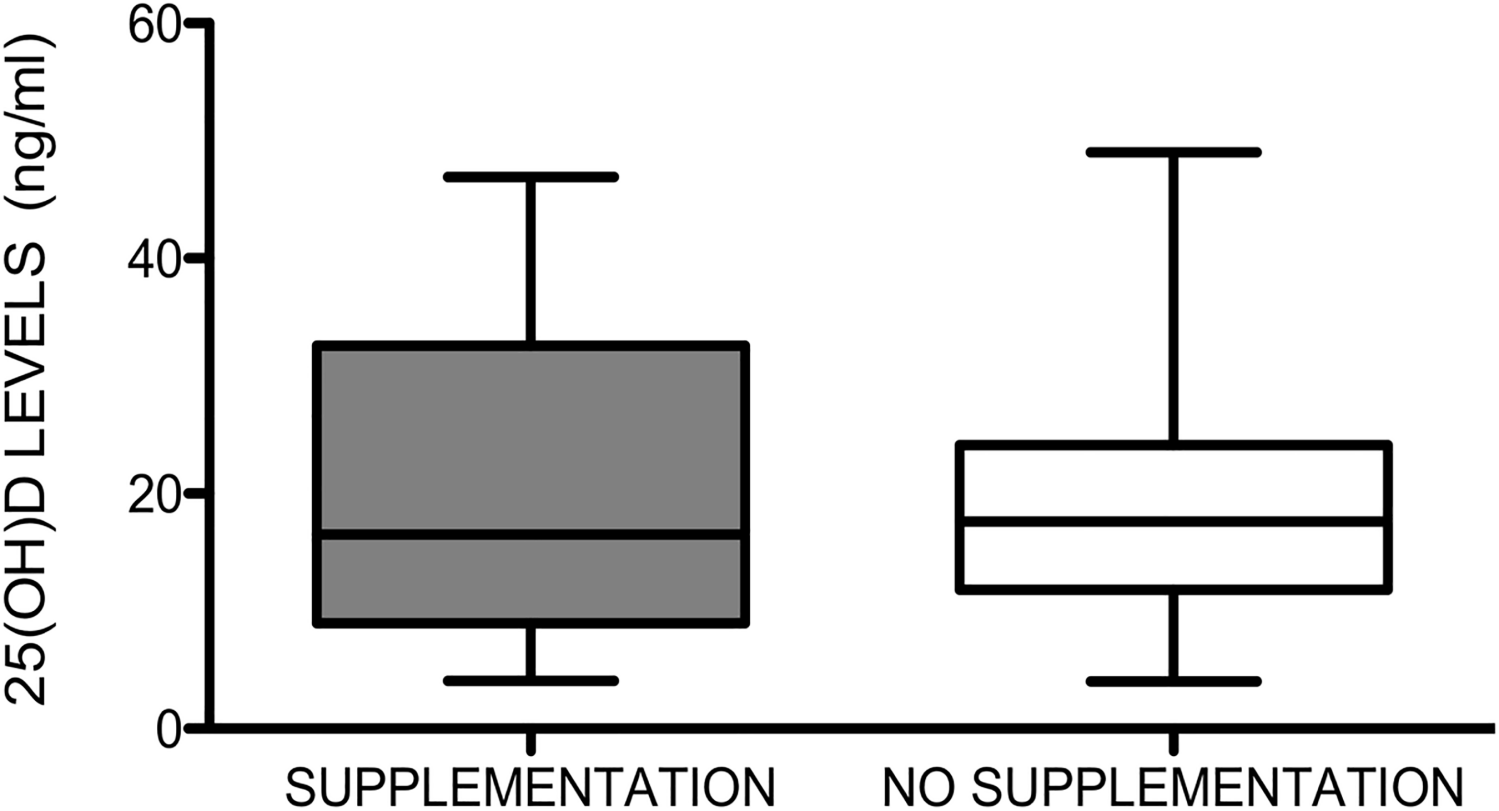
25(OH)D serum concentrations and treatment. 25(OH)D serum concentrations compared in patients assuming/not assuming supplementation with 1,000 IU daily for at least 6–12 months. There was no influence of treatments with oral colecalciferol on 25(OH)D serum concentrations: 18.8 ±10 ng/ml in treated and 18.7 ±9 ng/ml in untreated patients (p = 0.81).

Relevant treatment regimens are reported in Table 1. Interestingly, there was no influence of treatments with oral colecalciferol (1,000 IU daily for 6–12 months) on 25(OH)D serum concentrations: 18.8 ±10 ng/ml in treated and 18.7 ±9 ng/ml in untreated patients (p = 0.81) (Fig 3).

No difference in 25(OH)D serum concentrations were observed in patients assuming/not assuming glucocorticoids (21 ±12 ng/ml VS 17.8 ±9 ng/ml respectively; p = 0.36), cyclic intravenous iloprost (19 ±9 ng/ml VS 18 ± 10 ng/ml; p = 0.43), calcium channel blockers (22 ±11 ng/ml VS 17.9 ±9 ng/ml respectively; p = 0.09), pulmonary arterial hypertension (ERAs) treatment (19 ± 10 ng/ml VS 18 ±10 ng/ml, p = 0.5) (Table 1).

## Discussion

Serum 25(OH)D deficiency was found in more than 50% of SSc patients and significant fluctuations according to seasons were confirmed in almost all patients. In addition, for the first time to our knowledge, deficent/insufficient 25(OH)D serum concentrations were found to be independent from standard oral supplementation.

As a matter of fact, 25(OH)D serum concentrations were below 30 ng/ml in more then 80% of the SSc patients. This reported cut-off level for “sufficiency” was established because intestinal calcium absorption is optimized at a concentration above 30–32 ng/ml and parathyroid hormone start to rise when 25(OH)D is below 31 ng/ml [15].

As expected, calcium levels correlated almost significantly with 25(OH)D serum concentrations, conversely we found a non significant increase in PTH levels, but probably these data are affected by the smaller number of patients examined.

The variability of 25(OH)D serum concentrations was demonstrated to have a strong genetic component [16]. A good example is SLE, in which serum 25(OH)D serum concentrations were associated with vitamin D receptor (VDR) polymorphisms [17].

A recent study showed that low serum concentrations of 25(OH)D has negative impact in diffuse SSc patients’ quality of life and in addition, a correlation with severe NVC alterations was reported, suggesting a possible role of 25(OH)D in vascular involvement [18]. Furthermore, recently a significant difference in serum IgM anti-vitamin D antibodies in SSc patients was found, compared to healthy controls [19]. Other Authors did not find correlations between a more severe disease and 25(OH)D serum concentrations [20].

We have shown in the present large study from two European referral centers, that low 25(OH)D serum concentrations are associated, in SSc patients, with bilateral fibrosis at lung CT scan and to Medsger’s DSS parameters “peripheral vascular”, “kidney” and “gastrointestinal”.

A severe lung involvement was already described in SSc patients with low 25(OH)D serum concentrations [21]. In the present study however, we do not observe a linear correlation between 25(OH)D concentration and DLCO values, but only a more severe 25(OH)D deficiency was observed in patients with bilateral pulmonary fibrosis on lung CT scan compared to those with milder/no lung involvement. Therefore, in this case hypovitaminosis D seems to be associated only to severe pulmonary structural damage.

The scarce significance of data on gut and lung involvement is probably due to the pronounced 25(OH)D deficiency in all patients. Furthermore, this particular population did not show a great differentiation in terms of gastrointestinal involvement according to Medsger’s DSS, having very few patients in classes 3 and 4.

Regarding Medsger’s DSS “peripheral vascular” parameter, this is based on both severity of Raynaud’s phenomenon and presence of digital lesions (0 = normal, 1 = Raynaud’s requiring vasodilators, 2 = digital pitting scars, 3 = digital tip ulcerations, 4 = digital gangrene) [9]. In the study population, DUs incidence (stage 3 of “peripheral vascular parameter”) taken singularly or presence of any digital trophic lesion (stage 2 to 4 of “peripheral vascular parameter”), appeared to correlate strongly with NVC patterns of microangiopathy and not with 25(OH)D serum concentrations. Our data confirm literature reports affirming that patients with “Late” NVC pattern are more prone to development of trophic digital lesions [1]. Probably for this parameter, only degrees 0 and 1 of Medsger’s DSS are more related to functional/milder alterations of the vascular system, mostly influencing the lack of vitamin D efficient metabolism.

Organ involvement and disease severity relation with vitamin D, could be explained by its immune-regolatory properties. In fact, impairment of self-tolerance and immune responses, through altered regulation of dendritic cells, regulatory T-lymphocytes (Tregs), Th1 cells and B cells functions were described in hypovitaminosis D [22]. Even in healthy controls, antinuclear antibodies (ANA)-positivity was associated with lower vitamin D serum concentration in comparison with ANA negative individuals [23].

Previous studies demonstrated that, in rheumatoid arthritis synovial tissue, 1,25-dihydroxyvitamin D3 may downregulate pro-inflammatory cytokine production in activated macrophages, decreasing aromatase activity, especially in presence of an estrogenic milieu [24]. A study from our group showed, for the first time, in 53 SSc patients and 35 healthy controls, the significant seasonal changes of 25(OH)D serum concentrations, with peak values in late summer and decreased values in late winter [25].

An important finding of this study is that a seasonal variation in 25(OH)D serum concentration is confirmed. The result would suggest that vitamin D synthesis phases held in the skin are at least partially preserved. This concept might have an interest for the development of therapeutic strategies for hypovitaminosis D in SSc patients.

Ultraviolet B rays (UVB) cause the non-enzymatic conversion of 7-dehydrocholesterol to pre-vitamin D3 In the skin. In the liver, pre-vitamin D3 is converted in 25(OH)D3 and then to the active form 1,25-dihydroxivitamin D3 in the kidney. For this reason, the first step for adequate vitamin D supply to the human body is through adequate skin synthesis.

It is possible that in SSc patients with severe organ involvement, sunlight exposure could be reduced for different reasons (sedentary attitude, reduced mobility, hospitalization) contributing to the high prevalence of vitamin D deficiency/insufficiency. Moreover, the higher skin pigmentation described in SSc patients is a factor to be considered, especially in those affected by diffuse form [26]. In fact, cutaneous pre-vitamin D production was described to be related to melanin amount in the skin, being melanin a good absorbent of UVB rays [27]. Higher skin pigmentation was demonstrated to worsen vitamin D deficiency, as well as malabsorption and small intestine function is known to be frequently altered in SSc patients [28–31].

However, independently from severity of the disease, some patients with malabsorption have a normal concentration of serum vitamin D, while healthy subjects can show hypovitaminosis [32]. A common factor in SSc, Crohn’s disease and ulcerative colitis that may be a contributory cause for vitamin D deficit is intestinal inflammation [33]. Fecal calprotectin is a common marker of gut involvement in all conditions cited and was shown to correlate independently with low vitamin D serum concentration in SSc patients [34]. Furthermore, gut is one of the extra-renal sites were inflammation drives the hyper-expression of CYP24A1 and CYP27B1, therefore, reducing 25(OH)D through induction of a higher rate of conversion to 1,25(OH)_2_D.

Reduced absorption capacity of small intestine was postulated to be determinant for failure of oral treatment with physiological/standard doses of oral colecalciferol [35].

Vitamin D3 (colecalciferol, Dibase Abiogen Pharma, Italy) 1,000 IU/die was used in our patients for oral supplementation, being its effectiveness already demonstrated in comparison with vitamin D2 [36]. In a 2014 Cochrane Review, the Authors concluded that vitamin D3 seemed to decrease mortality in elderly people living independently or in institutional care while vitamin D2, alfacalcidol and calcitriol had no statistically significant beneficial effects on mortality [37]. Interestingly, in our patients population, the oral substitutive treatment with oral colecalciferol (time range 6–12 months) was not influencing 25(OH)D serum concentration, while an evident influence was exerted by seasons, with patients observed in winter months having a significantly more severe hypovitaminosis D, compared to those observed in summer months. The result seems coherent with reports on vitamin D deficiency secondary to diseases characterized by malabsorption [38, 39]. However, some authors postulate the possibility of achieving better results in selected patients using vitamin supra-physiological vitamin D doses: up to 5,000 IU daily for 24 weeks for three-six months in Chron’s disease, 50,000 IU bi-weekly for 12 weeks in cystic fibrosis were used [40, 41].

Sensible sun exposure, especially between the hours of 10:00 am and 3:00 pm seems to produce serum 25(OH)D in the skin lasting twice as long compared with vitamin D assumed orally. However, as mentioned, a variety of factors reduce the skin production, including increased skin pigmentation, aging, and topical application of a sunscreen [42].

A clinical study from Sweden showed UVB therapy to be more efficacious in raising serum 25(OH)D concentrations in deficient subjects. This suggests that UVB therapy may be a useful therapeutic approach in selected individuals [43].

It has to be mentioned that new *in vivo* pathways of D3 metabolism regulated by P450 and CYP11A1 cytochromes were described, in recent years, in animals and humans steroidogenic tissues. Products of the aforementioned metabolisms are D3 hydroxyderivatives (main metabolite 20(OH)D3) with activities that are similar to 25(OH)D3 and 1,25(OH)2D3 [44–46]. The Authors demonstrated that human dermal fibroblasts can transform D3 to 20(OH)D3, 22(OH)D3, 20,22(OH)2D3, 20,23(OH)2D3 and 1,20(OH)2D3 [47]. Therefore, P450 and CYP11A1-derived secosteroids might be studied, in the future, in diseases involving the skin, for their pathogenic and therapeutic potentials.

This study had several limitations. We did not have a control group but we compared 25(OH)D serum concentration with average healthy population values. However it as to be said that it was not among the purposes of this study to demonstrate a difference in 25(OH)D serum concentrations between SSc patients and healthy subjects, being this topic already addressed by other Authors [2, 3]. We mainly wanted to investigate on the possible existing relations between 25(OH)D serum concentrations, clinical complications, seasonality and supplementation with oral colecalciferol.

In addition, we did not evaluate PTH serum concentrations in all patients and in no patient serum concentration of 1,25(OH)_2_D were performed. However, the 25(OH)D deficit has been reported to be due to higher conversion rate of 25(OH)D to 1,25(OH)2D upon extra-renal hyperactivity of the CYP27B1 in inflamed tissues [48].

Since this was a retrospective data analysis, we have not had the opportunity to perform other tests to assess the severity of a possible malabsorption (iron or other vitamins deficiency, elettrolites alterations, hypo-proteinemia, hypo-lipemia, stool examination alterations).

Finally, precise quantitative reports of sunlight exposure and cutaneous hyperpigmentation in patients were not reported.

In conclusion, a serum 25(OH)D deficiency in SSc is confirmed. The last seems to be worse in cold seasons and to correlate with severe lung involvement at CT scan, as well as with “peripheral vascular”, “kidney” and “gastrointestinal” damage (Medsger’s DSS). Low 25(OH)D serum concentrations failed to be corrected by oral colecalciferol administration. Therefore, we suggest to monitor 25(OH)D serum concentrations after 3–6 months from the start of treatment. In case of persistently low 25(OH)D serum concentrations, supra-physiological oral vitamin D doses or programmed UVB light exposure should be considered [49, 50].

## Supporting information

S1 File Patients data. (XLS)

## References

[1] Cutolo M, Sulli A, Smith V. Assessing microvascular changes in systemic sclerosis diagnosis and management. Nat Rev Rheumatol 2010;6:578–87. doi: 10.1038/nrrheum.2010.104 20703220 10.1038/nrrheum.2010.104

[2] Vacca A, Cormier C, Piras M, Mathieu A, Kahan A, Allanore Y. Vitamin D deficiency and insufficiency in 2 independent cohorts of patients with systemic sclerosis. J Rheumatol 2009;36:1924–9. doi: 10.3899/jrheum.081287 19648299 10.3899/jrheum.081287

[3] Gambichler T, Chrobok I, Höxtermann S, Kreuter A. Significantly decreased serum 25-hydroxyvitamin d levels in a large German systemic sclerosis cohort. J Rheumatol 2011;38:2492–3. doi: 10.3899/jrheum.110695 22045936 10.3899/jrheum.110695

[4] Cutolo M, Otsa K, Uprus M, Paolino S, Seriolo B. Vitamin D in rheumatoid arthritis. Autoimmun Rev 2007;7:59–64. doi: 10.1016/j.autrev.2007.07.001 17967727 10.1016/j.autrev.2007.07.001

[5] Linker-Israeli M, Elstner E, Klinenberg JR, Wallace DJ, Koeffler HP. Vitamin D and its synthetic analogs inhibit the spontaneous in vitro immunoglobulin production by SLE-derived PBMC. Clin Immunol 2001;99:82–93. doi: 10.1006/clim.2000.4998 11286544 10.1006/clim.2000.4998

[6] Arnson Y, Amital H, Agmon-Levin N, Alon D, Sánchez-Castañón M, López-Hoyos M et al Serum 25-OH vitamin D concentrations are linked with various clinical aspects in patients with systemic sclerosis: a retrospective cohort study and review of the literature. Autoimmun Rev 2011;10:490–4. doi: 10.1016/j.autrev.2011.02.002 21320645 10.1016/j.autrev.2011.02.002

[7] LeRoy EC, Medsger TA Jr. Criteria for the classification of early systemic sclerosis. J Rheumatol. 2001;28:1573–6. 11469464

[8] van den Hoogen F, Khanna D, Fransen J, Johnson SR, Baron M, Tyndall A, et al 2013 classification criteria for systemic sclerosis: an American college of rheumatology/European league against rheumatism collaborative initiative. Ann Rheum Dis. 2013;72:1747–55. doi: 10.1136/annrheumdis-2013-204424 24092682 10.1136/annrheumdis-2013-204424

[9] Medsger TA Jr, Silman AJ, Steen VD, Black CM, Akesson A, Bacon PA, et al A disease severity scale for systemic sclerosis: development and testing. J Rheumatol 1999;26:2159–67. 10529133

[10] Hollis BW, Wagner CL. Normal serum vitamin D levels. N Engl J Med 2005;352:515–6. 10.1056/NEJM200502033520521 15689596

[11] Sulli A, Secchi ME, Pizzorni C, Cutolo M. Scoring the nailfold microvascular changes during the capillaroscopic analysis in systemic sclerosis patients. Ann Rheum Dis 2008;67:885–7. doi: 10.1136/ard.2007.079756 18037628 10.1136/ard.2007.079756

[12] Cutolo M1, Pizzorni C, Secchi ME, Sulli A. Capillaroscopy. Best Pract Res Clin Rheumatol 2008;22:1093–108. doi: 10.1016/j.berh.2008.09.001 19041079 10.1016/j.berh.2008.09.001

[13] Cutolo M1, Sulli A, Pizzorni C, Accardo S. Nailfold videocapillaroscopy assessment of microvascular damage in systemic sclerosis. J Rheumatol 2000;27:155–60. 10648032

[14] Gosfield E 3rd, Bonner FJ Jr. Evaluating bone mineral density in osteoporosis. Am J Phys Med Rehabil 2000; 79:283–91. 10821315 10.1097/00002060-200005000-00011

[15] Heaney RP, Dowell MS, Hale CA, Bendich A. Calcium absorption varies within the reference range for serum 25-hydroxyvitamin D. J Am Coll Nutr. 2003;22:142–6. 12672710 10.1080/07315724.2003.10719287

[16] Livshits G, Karasik D, Seibel MJ. Statistical genetic analysis of plasma levels of vitamin D: familial study. Ann Hum Genet 1999;63:429–39. 10735584 10.1046/j.1469-1809.1999.6350429.x

[17] Monticielo OA, Brenol JC, Chies JA, Longo MG, Rucatti GG, Scalco R, et al The role of BsmI and FokI. Vitamin D receptor gene polymorphisms and serum 25-hydroxyvitamin D in Brazilian patients with systemic lupus erythematosus. Lupus 2012;21:43–52. doi: 10.1177/0961203311421798 21993390 10.1177/0961203311421798

[18] Sampaio-Barros MM, Takayama L, Sampaio-Barros PD, Bonfá E, Pereira RM. Low vitamin D serum levels in diffuse systemic sclerosis: a correlation with worst quality of life and severe capillaroscopic findings. Rev Bras Reumatol Engl Ed 2016;56:337–44. doi: 10.1016/j.rbre.2016.05.006 27476627 10.1016/j.rbre.2016.05.006

[19] Carmel NN, Rotman-Pikielny P, Lavrov A, Levy Y. Vitamin D Antibodies in Systemic Sclerosis Patients: Findings and Clinical Correlations Isr Med Assoc J. 2015;17:80–4. 26223082

[20] Belloli L, Ughi N, Marasini B. Vitamin D in systemic sclerosis. Clin Rheumatol 2011;30:145–6. doi: 10.1007/s10067-010-1564-6 20878341 10.1007/s10067-010-1564-6

[21] Groseanu L, Bojinca V, Gudu T, Saulescu I, Predeteanu D, Balanescu A, et al Low vitamin D status in systemic sclerosis and the impact on disease phenotype Eur J Rheumatol 2016;3:50–55. doi: 10.5152/eurjrheum.2015.0065 27708971 10.5152/eurjrheum.2015.0065 PMC5042230

[22] Cutolo M, Plebani M, Shoenfeld Y, Adorini L, Tincani A. Vitamin D Endocrine System and the Immune Response in Rheumatic Diseases Vitam Horm 2011;86:327–51. doi: 10.1016/B978-0-12-386960-9.00014-9 21419278 10.1016/B978-0-12-386960-9.00014-9

[23] Ritterhouse LL, Crowe SR, Niewold TB, Kamen DL, Macwana SR, Roberts VC, et al Vitamin D deficiency is associated with an increased autoimmune response in healthy individuals and in patients with systemic lupus erythematosus. Ann Rheum Dis 2011;70:1569–74. doi: 10.1136/ard.2010.148494 21586442 10.1136/ard.2010.148494 PMC3149865

[24] Villaggio B, Soldano S, Cutolo M. 1,25-dihydroxyvitamin D3 downregulates aromatase expression and inflammatory cytokines in human macrophages. Clin Exp Rheumatol 2012;30:934–8. 23253631

[25] Seriolo B, Molfetta L, Cutolo M. Seasonal variations in serum levels of 25-hydroxyvitamin D in patients with systemic sclerosis. Clin Rheumatol 2011;30:445–6. doi: 10.1007/s10067-011-1684-7 21234627 10.1007/s10067-011-1684-7

[26] Pope JE, Shum DT, Gottschalk R, Stevens A, McManus R. Increased pigmentation in scleroderma. J Rheumatol 1996;23:1912–6. 8923365

[27] Clemens TL, Adams JS, Henderson SL, Holick MF. Increased skin pigment reduces the capacity of skin to synthesise vitamin D3. Lancet 1982;1:74–6. 6119494 10.1016/s0140-6736(82)90214-8

[28] Fu YT, Chatur N, Cheong-Lee C, Salh B. Hypovitaminosis D in adults with inflammatory bowel disease: potential role of ethnicity. Dig Dis Sci 2012;57:2144–8. doi: 10.1007/s10620-012-2130-7 22451117 10.1007/s10620-012-2130-7

[29] Nishikawa J, Mihara H, Sugiyama T. Systemic sclerosis in small bowel. Clin Gastroenterol Hepatol. 2013;11:A21. 10.1016/j.cgh.2012.08.036 22982099

[30] Tavakkoli A, DiGiacomo D, Green PH, Lebwohl B. Vitamin D status and concomitant autoimmunity in celiac disease. J Clin Gastroenterol 2013;47:515–9. doi: 10.1097/MCG.0b013e318266f81b 23328299 10.1097/MCG.0b013e318266f81b PMC3640651

[31] Wolfenden LL, Judd SE, Shah R, Sanyal R, Ziegler TR, Tangpricha V. Vitamin D and bone health in adults with cystic fibrosis. Clin Endocrinol (Oxf) 2008;69:374–81. 18284636 10.1111/j.1365-2265.2008.03216.x PMC2851223

[32] Margulies SL, Kurian D, Elliott MS, Han Z. Vitamin D deficiency in patients with intestinal malabsorption syndromes—think in and outside the gut. J Dig Dis 2015;16:617–33. doi: 10.1111/1751-2980.12283 26316334 10.1111/1751-2980.12283

[33] Garg M, Rosella O, Lubel JS, Gibson PR. Association of circulating vitamin D concentrations with intestinal but not systemic inflammation in inflammatory bowel disease. Inflamm Bowel Dis 2013;19:2634–43. doi: 10.1097/01.MIB.0000436957.77533.b2 24105392 10.1097/01.MIB.0000436957.77533.b2

[34] Marie I, Leroi AM, Menard JF, Levesque H, Quillard M, Ducrotte P. Fecal calprotectin in systemic sclerosis and review of the literature. Autoimmun Rev 2015;14:547–54. doi: 10.1016/j.autrev.2015.01.018 25661980 10.1016/j.autrev.2015.01.018

[35] Robberecht E, Vandewalle S, Wehlou C, Kaufman JM, De Schepper J. Sunlight is an important determinant of vitamin D serum concentrations in cystic fibrosis. Eur J Clin Nutr 2011;65:574–9. doi: 10.1038/ejcn.2010.280 21245888 10.1038/ejcn.2010.280

[36] Logan VF, Gray AR, Peddie MC, Harper MJ, Houghton LA. Long-term vitamin D3 supplementation is more effective than vitamin D2 in maintaining serum 25-hydroxyvitamin D status over the winter months. Br J Nutr 2013;109:1082–8. doi: 10.1017/S0007114512002851 23168298 10.1017/S0007114512002851

[37] Bjelakovic G, Gluud LL, Nikolova D, Whitfield K, Wetterslev J, Simonetti RG, et al Vitamin D supplementation for prevention of mortality in adults. Cochrane Database Syst Rev 2014 10;(1):CD007470. 10.1002/14651858.CD007470.pub3 PMC11285307 24414552

[38] Suibhne TN, Cox G, Healy M, O’Morain C, O’Sullivan M. Vitamin D deficiency in Crohn’s disease: prevalence, risk factors and supplement use in an outpatient setting. J Crohns Colitis 2012;6:182–8. doi: 10.1016/j.crohns.2011.08.002 22325172 10.1016/j.crohns.2011.08.002

[39] McCarthy D, Duggan P, O'Brien M, Kiely M, McCarthy J, Shanahan F, et al Seasonality of vitamin D status and bone turnover in patients with Crohn’s disease. Aliment Pharmacol Ther 2005;21:1073–83. doi: 10.1111/j.1365-2036.2005.02446.x 15854168 10.1111/j.1365-2036.2005.02446.x

[40] Pappa HM, Mitchell PD, Jiang H et al Treatment of vitamin D insufficiency in children and adolescents with inflammatory bowel disease: a randomized clinical trial comparing three regimens. J Clin Endocrinol Metab 2012;97:2134–42. doi: 10.1210/jc.2011-3182 22456619 10.1210/jc.2011-3182 PMC3387426

[41] Hall WB, Sparks AA, Aris RM. Vitamin D deficiency in cystic fibrosis. Int J Endocrinol 2010;2010:218691 doi: 10.1155/2010/218691 20148079 10.1155/2010/218691 PMC2817861

[42] Haddad JG; Matsuoka LY; Hollis BW; Hu YZ; Wortsman J. Human plasma transport of vitamin D after its endogenous synthesis. J Clin Invest 1993;91:2552–5. doi: 10.1172/JCI116492 8390483 10.1172/JCI116492 PMC443317

[43] Bogh MK, Gullstrand J, Svensson A, Ljunggren B, Dorkhan M. Narrowband ultraviolet B three times per week is more effective in treating vitamin D deficiency than 1600 IU oral vitamin D₃ per day: a randomized clinical trial. Br J Dermatol 2012;167:625–30.50. doi: 10.1111/j.1365-2133.2012.11069.x 22632734 10.1111/j.1365-2133.2012.11069.x

[44] Slominski AT, Kim TK, Shehabi HZ, Semak I, Tang EK, Nguyen MN, et al In vivo evidence for a novel pathway of vitamin D₃ metabolism initiated by P450scc and modified by CYP27B1. FASEB J. 2012;26:3901–15. doi: 10.1096/fj.12-208975 22683847 10.1096/fj.12-208975 PMC3425822

[45] Slominski AT, Kim TK, Li W, Postlethwaite A, Tieu EW, Tang EK6, et al. Detection of novel CYP11A1-derived secosteroids in the human epidermis and serum and pig adrenal gland. Sci Rep. 2015 10 8;5:14875 doi: 10.1038/srep14875 26445902 10.1038/srep14875 PMC4597207

[46] Slominski AT1, Li W2, Kim TK3, Semak I4, Wang J2, Zjawiony JK et al Novel activities of CYP11A1 and their potential physiological significance. J Steroid Biochem Mol Biol 2015 7;151:25–37. doi: 10.1016/j.jsbmb.2014.11.010 25448732 10.1016/j.jsbmb.2014.11.010 PMC4757911

[47] Slominski AT, Kim TK, Li W, Tuckey RC. Classical and non-classical metabolic transformation of vitamin D in dermal fibroblasts. Exp Dermatol. 2016 3;25:231–2. doi: 10.1111/exd.12872 26440881 10.1111/exd.12872 PMC4789164

[48] Abreu MT1, Kantorovich V, Vasiliauskas EA, Gruntmanis U, Matuk R, Daigle K, et al Measurement of vitamin D levels in inflammatory bowel disease patients reveals a subset of Crohn’s disease patients with elevated 1,25-dihydroxyvitamin D and low bone mineral density. Gut 2004;53:1129–36. doi: 10.1136/gut.2003.036657 15247180 10.1136/gut.2003.036657 PMC1774134

[49] Koutkia P, Lu Z, Chen TC, Holick MF. Treatment of vitamin D due to Crohn’s disease with tanning bed ultraviolet B radiation. Gastroenterology 2001; 121: 1485–8. 11729127 10.1053/gast.2001.29686

[50] Tangpricha V, Kelly A, Stephenson A, Maguiness K, Enders J, Robinson KA, et al An update on the screening, diagnosis, management, and treatment of vitamin D deficiency in individuals with cystic fibrosis: evidence-based recommendations from the Cystic Fibrosis Foundation. J Clin Endocrinol Metab 2012;97:1082–93. doi: 10.1210/jc.2011-3050 22399505 10.1210/jc.2011-3050

